# Estimating retention benchmarks for salvage logging to protect biodiversity

**DOI:** 10.1038/s41467-020-18612-4

**Published:** 2020-09-21

**Authors:** Simon Thorn, Anne Chao, Kostadin B. Georgiev, Jörg Müller, Claus Bässler, John L. Campbell, Jorge Castro, Yan-Han Chen, Chang-Yong Choi, Tyler P. Cobb, Daniel C. Donato, Ewa Durska, Ellen Macdonald, Heike Feldhaar, Joseph B. Fontaine, Paula J. Fornwalt, Raquel María Hernández Hernández, Richard L. Hutto, Matti Koivula, Eun-Jae Lee, David Lindenmayer, Grzegorz Mikusiński, Martin K. Obrist, Michal Perlík, Josep Rost, Kaysandra Waldron, Beat Wermelinger, Ingmar Weiß, Michał Żmihorski, Alexandro B. Leverkus

**Affiliations:** 1 Field Station Fabrikschleichach, Biocenter, University of Würzburg, Glashüttenstr. 5, 96181 Rauhenebrach, Germany; 2 Institute of Statistics, National Tsing Hua University, Hsin-Chu, 30043 Taiwan; 3 Bavarian Forest National Park, Freyunger Str. 2, 94481 Grafenau, Germany; 4 Department of Biodiversity Conservation, Goethe University Frankfurt, Faculty of Biological Sciences, Institute for Ecology, Evolution and Diversity, Max-von-Laue-Str. 13, D-60438 Frankfurt am Main, Germany; 5 Department of Forest Ecosystems and Society, Oregon State University, 321 Richardson Hall, Corvallis, OR 97331 USA; 6 Department of Ecology, University of Granada, Campus Fuentenueva s/n, 18071 Granada, Spain; 7 Department of Agriculture, Forestry, and Bioresources, Seoul National University, Seoul, 08826 Korea; 8 Royal Alberta Museum, Edmonton, AB T5J 0G2 Canada; 9 School of Environmental and Forest Sciences, University of Washington, Seattle, WA 98195 USA; 10 Museum and Institute of Zoology, Polish Academy of Sciences, Wilcza 64, 00-679 Warsaw, Poland; 11 Department of Renewable Resources, University of Alberta, Edmonton, AB T6G 2H1 Canada; 12 Department of Animal Ecology I, Bayreuth Center for Ecology and Environmental Research (BayCEER), University of Bayreuth, 95447 Bayreuth, Germany; 13 Environmental and Conservation Sciences, Murdoch University, 90 South Street, Murdoch, WA 6150 Australia; 14 USDA Forest Service, Rocky Mountain Research Station, 240 West Prospect Road, Fort Collins, CO 80526 USA; 15 Department of Botany, Ecology and Plant Physiology, Universidad de La Laguna, 38200 San Cristóbal de La Laguna, Spain; 16 Division of Biological Sciences, University of Montana, Missoula, MT 59812 USA; 17 Natural Resources Institute (LUKE), P. O. Box 2, FI-00791 Helsinki, Finland; 18 Urban Planning Research Group, Daejeon Sejong Research Institute, Daejeon, 34863 Korea; 19 Fenner School of Environment and Society, The Australian National University, Canberra, ACT 2601 Australia; 20 Grimsö Wildlife Research Station, Department of Ecology, Swedish University of Agricultural Sciences SLU, SE-730 91 Riddarhyttan, Sweden; 21 School for Forest Management, Swedish University of Agricultural Sciences SLU, Box 43, SE-739 21 Skinnskatteberg, Sweden; 22 WSL Swiss Federal Institute for Forest, Snow and Landscape Research, Biodiversity and Conservation Biology, Zürcherstrasse 111, CH-8903 Birmensdorf, Switzerland; 23 Faculty of Science, University of South Bohemia, Branisovska 1760, 37005 Ceske Budejovice, Czech Republic; 24 Institute of Entomology, Biology Centre of the Czech Academy of Sciences, Branisovska 31, 37005 Ceske Budejovice, Czech Republic; 25 Department of Environmental Sciences, University of Girona. Facultat de Ciències, Carrer Maria Aurèlia Capmany, Campus de Montilivi, 17003 Girona, Catalonia Spain; 26 Natural Resources Canada, Canadian Forest Service, Laurentian Forestry Centre, 1055 rue du P.E.P.S., P.O. Box 10380, Stn. Sainte-Foy, Québec, QC G1V 4C7 Canada; 27 WSL Swiss Federal Institute for Forest, Snow and Landscape Research, Forest Health and Biotic Interactions-Forest Entomology, Zürcherstrasse 111, CH-8903 Birmensdorf, Switzerland; 28 Rehtränke, 94481 Rosenau, Germany; 29 Mammal Research Institute, Polish Academy of Sciences, Stoczek 1, 17-230 Białowieża, Poland

**Keywords:** Biodiversity, Forest ecology, Forestry, Environmental impact

## Abstract

Forests are increasingly affected by natural disturbances. Subsequent salvage logging, a widespread management practice conducted predominantly to recover economic capital, produces further disturbance and impacts biodiversity worldwide. Hence, naturally disturbed forests are among the most threatened habitats in the world, with consequences for their associated biodiversity. However, there are no evidence-based benchmarks for the proportion of area of naturally disturbed forests to be excluded from salvage logging to conserve biodiversity. We apply a mixed rarefaction/extrapolation approach to a global multi-taxa dataset from disturbed forests, including birds, plants, insects and fungi, to close this gap. We find that 75 ± 7% (mean ± SD) of a naturally disturbed area of a forest needs to be left unlogged to maintain 90% richness of its unique species, whereas retaining 50% of a naturally disturbed forest unlogged maintains 73 ± 12% of its unique species richness. These values do not change with the time elapsed since disturbance but vary considerably among taxonomic groups.

## Abstract (web summary)

Salvage logging has become a common practice to gain economic returns from naturally disturbed forests, but it could have considerable negative effects on biodiversity. Here the authors use a recently developed statistical method to estimate that ca. 75% of the naturally disturbed forest should be left unlogged to maintain 90% of the species unique to the area.

## Introduction

The world’s forests are increasingly affected by natural disturbances, such as wildfires, windstorms or outbreaks of insect pests^1,2^. Increases in disturbance size, severity, and frequency are among the most severe impacts of climate change on forest ecosystems^3^. Many naturally disturbed forests are subsequently subjected to post-disturbance salvage logging, particularly in the temperate and boreal zones. Salvage logging is commonly justified to recover economic capital, reduce the risk of insect outbreaks, and decrease fire hazard^4^. It is sometimes also justified on the basis that it contributes to ecosystem recovery^5^. Salvage logging is conducted in all forest types, and is common even in areas that are otherwise excluded from logging, such as national parks^4^. By extracting timber and other tree biomass from large areas^5^, salvage logging can impair ecosystem services^6^ and affect the biodiversity of deadwood-dependent species^7^. Salvage logging can have more profound effects on biodiversity than natural disturbance or logging alone due to the additive and interacting effects of the two disturbances^8,9^. This has been exemplified by studies on changes in communities of birds^10–12^ and vascular plants^13^. Currently, unlogged early-successional forests following stand-replacing natural disturbances are among the most uncommon habitats in many regions of the world^14^. Not surprisingly, several species inhabiting these habitats have been targeted by conservation efforts. Examples include the black-backed woodpecker (*Picoides arcticus*) in the USA, largely restricted to burned forests and negatively affected by salvage logging;^15,16^ the tree fern (*Cyathea australis*) in Australia, present on disturbed sites but virtually eliminated from areas subject to salvage logging;^13^ and the white‐spotted sawyer beetle (*Monochamus scutellatus*) in Canada, present after single natural disturbances, but absent from salvage-logged forests^8^.

The increasing frequency and extent of natural disturbances have generated intense debates about the appropriateness of widespread, high-intensity salvage logging^17,18^. Hence, the retention of key structures in salvage logging operations (so-called biological legacies^19^), and the partial exclusion of naturally disturbed forests from salvage logging, are increasingly discussed as measures to halt the loss of forest biodiversity^7,20^. However, while benchmarks for a specific number of trees to be excluded from overall logging operations^21–23^ are common measures in modern retention forestry, such benchmarks are rare for salvage logging of naturally disturbed forests^24^. Existing guidelines for managing disturbed forest stands often recommend the complete removal of disturbance-killed trees, for instance of all disturbance-affected Norway spruce (*Picea abies*) exceeding 10 m³ per ha in Finland^25^. By contrast, recent recommendations^26^ advise the retention of all burned trees from fires larger than 100 ha in Catalonia, representing a minimum of 30% of all the burned area.

Estimating retention benchmarks for conserving biodiversity in the world’s naturally disturbed forests has been hampered by several factors^26^. First, the effects of salvage logging on alpha diversity of species vary widely among forest ecosystems and taxonomic groups, ranging from severe species losses in deadwood-dependent and forest-dwelling groups to increases in those species groups that prefer open habitats^7^. Second, studies based on comparing alpha diversity between logged and unlogged forests disregard the fact that assemblages found in any two distinct habitat patches may share a substantial fraction of species^27^. This regional diversity, which accumulates from compositional differences between local species assemblages (i.e., beta diversity), together with local alpha diversity, yields the overall gamma diversity in a study landscape^28^. Hence, net changes in species richness can mask changes in community composition caused by species losses and replacements^29^. This may, in turn, lead to biased estimates of retention benchmarks.

**Fig. 1:**
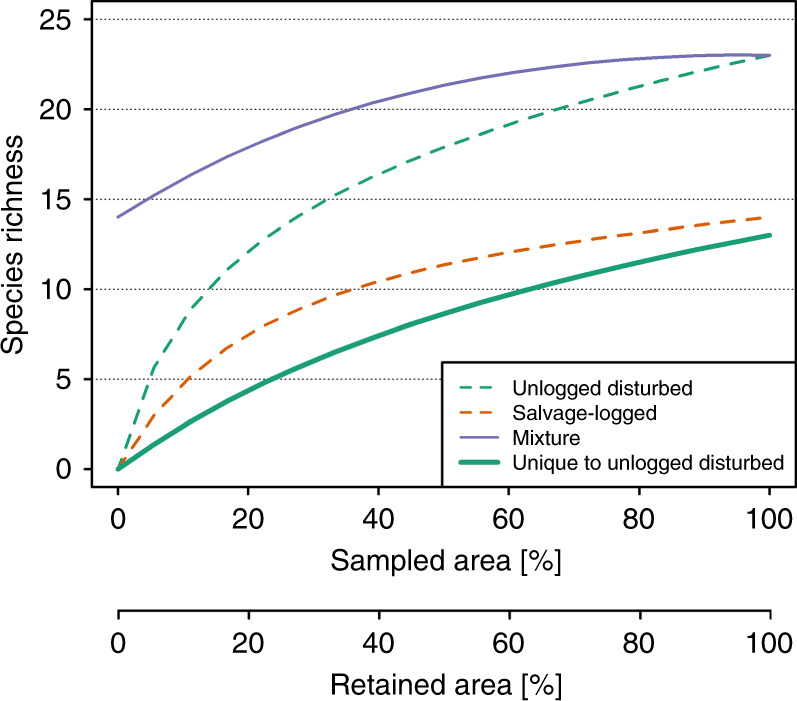
Hypothetical example of mixed rarefaction/extrapolation. The example depicts species sampled in naturally disturbed but unlogged plots and naturally disturbed and salvage-logged plots. Unlogged plots (dashed green curve, upper *x*-axis) had a higher species richness rarefaction curve than salvage-logged plots (dashed orange curve, upper x-axis) across the proportion of sampled area. The solid purple curve represents the species richness for a given mixture of salvage-logged and unlogged plots when a proportion of disturbed area remains unlogged (lower x-axis). The richness of species that are unique to unlogged plots (solid green curve, lower *x*-axis; used in our analysis) increases with increasing proportion of unlogged disturbed plots in the mixture. Here only a mixture of two rarefaction curves is presented; see ref. ^27^ for a mixture including both rarefaction and extrapolation curves.

We use a recently developed statistical approach based on a mixture of rarefaction and extrapolation to forecast changes in species richness when naturally disturbed forests are subjected to a successive transformation by salvage logging^27^. Our approach utilizes a proportional mixture of two within-habitat rarefaction/extrapolation curves to analytically predict biodiversity changes in landscapes when a specified proportion of an original habitat is transformed. In our approach, the two within-habitat rarefaction/extrapolation curves (Fig. 1, dashed curves) depict, respectively, the estimated species-area relationships for unlogged disturbed plots and salvage-logged plots. When a proportion of an unlogged disturbed area is salvage logged, the between-habitat compositional difference can be incorporated into the proportional mixture model to predict the resulting diversity change due to salvage logging (Fig. 1, solid purple curve).

The mixed rarefaction/extrapolation curve allows us to assess species richness for any mixture of two habitat types^27^ and to track the richness of species unique to unlogged, naturally disturbed forest (Fig. 1, bold green curve). These species are of high conservation interest and contribute greatly to community changes resulting from salvage logging^30^. We apply this statistical approach to a global dataset of studies with sampling units selected randomly from both naturally disturbed and salvage-logged areas to estimate logging benchmarks for naturally disturbed forests, namely a) the portion of naturally disturbed forest that must be spared from salvage logging to maintain 90% of the species richness associated with naturally disturbed and not salvage logged forest; and conversely b) the portion of species richness associated with naturally disturbed forest that remains when 50% of the area of a disturbed forest is salvage logged. Moreover, our statistical approach allows the quantification of species richness resulting from any portion of disturbed forest that is salvage logged.

We find that around 75% of a naturally disturbed area of a forest needs to be left unlogged to maintain 90% richness of its unique species, whereas retaining 50% of a disturbed forest unlogged maintains 73% of its unique species richness. These values, however, vary considerably among taxonomic groups, with deadwood-dependent (saproxylic) organisms, such as saproxylic beetles and wood-inhabiting fungi, generally requiring larger areas to be left unlogged than non-saproxylic taxa.

## Results

### Retention benchmarks

**Fig. 2:**
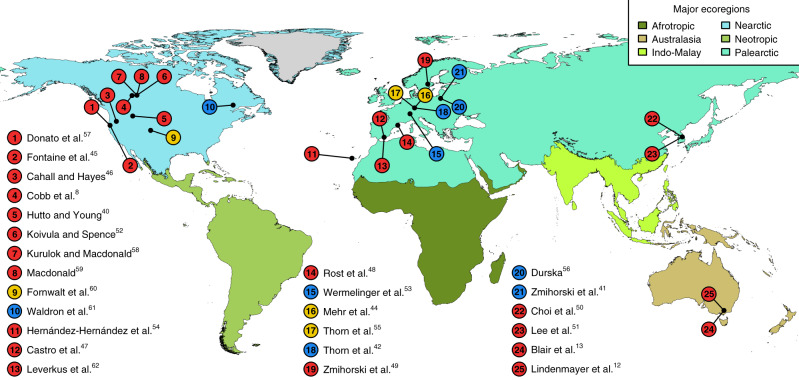
Location of studies included in the present analysis. Each study provided species-by-plot abundance matrices for salvage-logged and unlogged, naturally disturbed forest plots. Disturbance types are indicated by different symbol colors (red: wildfires, blue: windstorms, yellow: insect outbreaks; see Supplementary Table 1 for details and references). Background colors indicate major terrestrial ecoregions^67^.

We analyzed 201 full species-by-plot abundance matrices of 17 different taxonomic groups derived from 25 studies (Fig. 2).

**Fig. 3:**
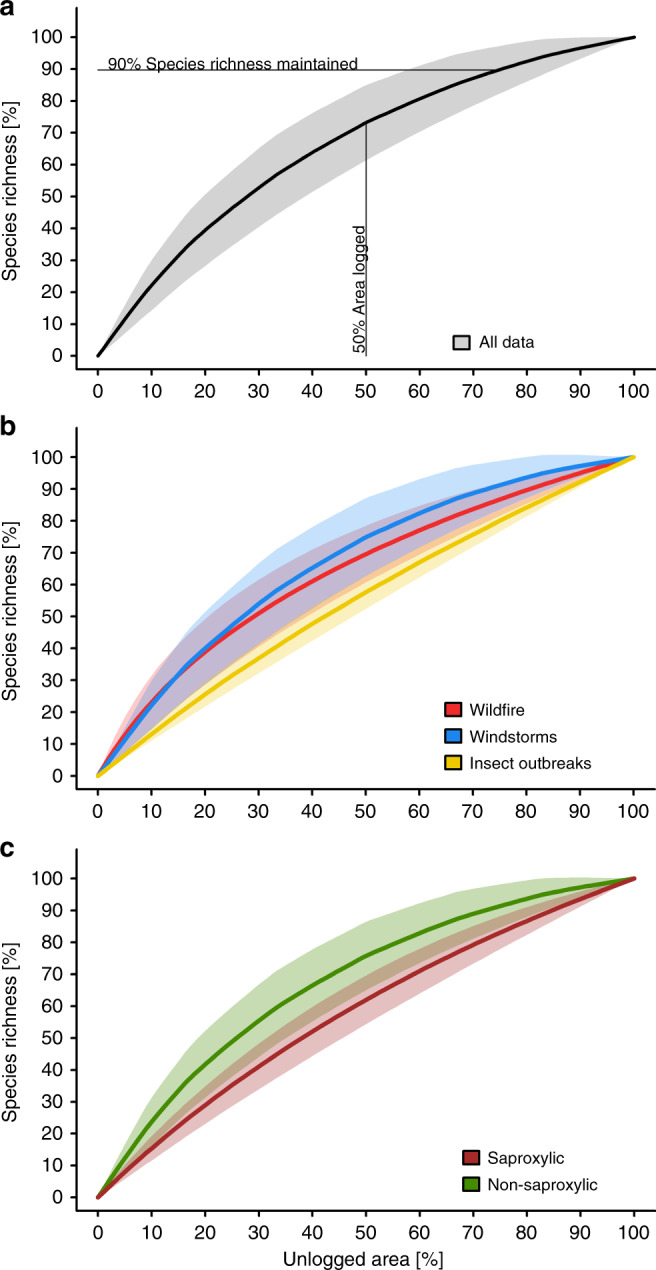
Response of species richness to different retention levels in salvage logging. Mean and standard deviation (shading) of richness of species unique to naturally disturbed, unlogged plots that would be maintained under varying portions of naturally disturbed forest excluded from salvage logging. The solid lines (means) are analogous to the solid green line from the hypothetical community in Fig. 1, indicating the mean response of 201 individual species matrices with (**a**) all data pooled, (**b**) datasets separated into different disturbance types, and (**c**) datasets separated into saproxylic and non-saproxylic taxa. Source data are provided as a Source Data file.

Averaging across all studies, our mixed rarefaction/extrapolation approach revealed that 75 ± 7% (mean ± SD) of a naturally disturbed area needs to be left unlogged to maintain 90% of the richness of species unique to it (Fig. 3a). These values ranged from a mean of 72 ± 8% in the case of windstorms to 81 ± 4% and 87 ± 2% for wildfires and insect outbreaks, respectively, with SD ranges largely overlapping (Fig. 3b). Saproxylic species groups needed, on average, larger areas to be retained (85 ± 3%) than non-saproxylic (72 ± 7%) species groups (Fig. 3c).

Salvage logging on 50% of the disturbed forest area reduced the richness of species unique to disturbed, unlogged forest to an average of 73 ± 12% (Fig. 3a). These values varied among disturbance types, and were lowest in insect-disturbed forests (reduction to 57 ± 5%), followed by burned forests (70 ± 9%), and wind-affected forests (75 ± 12%), with SD ranges largely overlapping (Fig. 3b). Species richness appeared most susceptible to subsequent salvage logging in insect-disturbed forests, although data were scant for this disturbance type. Saproxylic species suffered more than other species groups if 50% of the overall area was salvage logged, with species richness dropping to 61 ± 8% compared to unlogged forests. By contrast, non-saproxylic species groups dropped only to 75 ± 11% (Fig. 3c).

### Differences among taxonomic groups

**Fig. 4:**
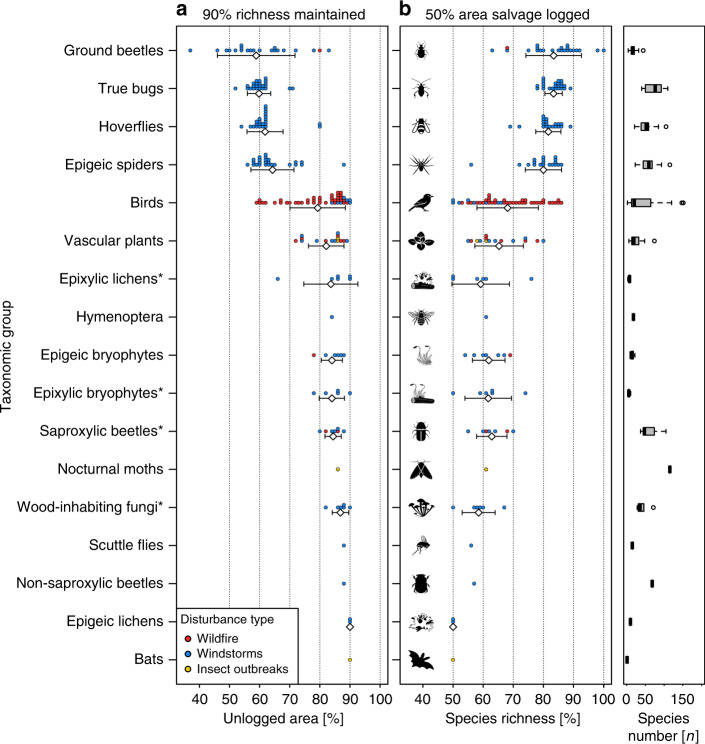
Estimated retention benchmarks for the assessed taxonomic groups. Distribution (dot histogram) and mean values (white diamonds) with corresponding standard deviation for: (**a**) the retention area needed to maintain 90% of species unique to unlogged naturally disturbed forest; and (**b**) the portion of species unique to unlogged naturally disturbed forest that would be maintained if 50% of the disturbed area was salvage logged. Saproxylic groups are marked with an asterisk. The right-hand box-whisker plots depict the median number of species unique to unlogged naturally disturbed plots (see Supplementary Table 1 for details) with lower and upper quartiles (box). Icons with permission from thenounproject.com. Source data are provided as a Source Data file.

The estimated proportion of a naturally disturbed area that needs to be left unlogged to maintain species richness varied considerably among taxonomic groups (Fig. 4). Preserving 90% of species richness unique to disturbed, unlogged forest of several saproxylic taxa, such as wood-inhabiting fungi, saproxylic beetles, and epixylic lichens, required that 80 to 90% of disturbed forest be retained. In contrast, preserving 90% of species richness unique to disturbed, unlogged forest of several non-saproxylic taxa, such as true bugs, ground beetles, hoverflies, and epigeic spiders, required 50 to 65% retention (Fig. 4a). Salvage logging of 50% of a naturally disturbed forest led to a decrease of around 60% of the original species richness unique to disturbed forest for several saproxylic taxa, such as epixylic lichens and wood-inhabiting fungi, and for vascular plants. In contrast, for species groups with large numbers of non-saproxylic species such as true bugs, hoverflies, epigeic spiders, and ground beetles, the richness of species unique to disturbed forest remained between 80 and 90% after salvage logging 50% of the disturbed area (Fig. 4b).

### Effect of time

**Fig. 5:**
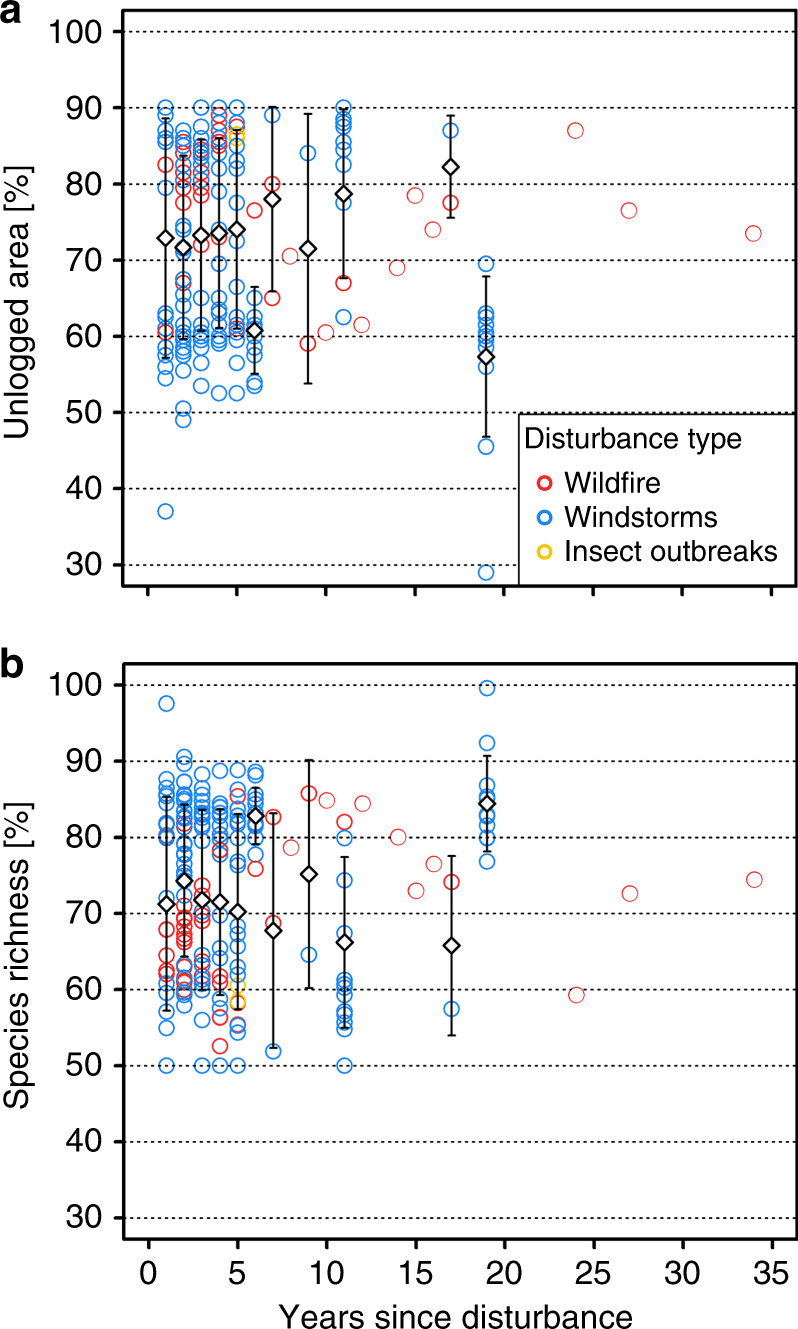
Response of retention benchmarks to time since disturbance. Distribution (scatterplot) and annual mean values (white diamonds) with corresponding standard deviation across years for: (**a**) the retention area needed to maintain 90% of species unique to unlogged naturally disturbed forest; and (**b**) the portion of species unique to unlogged naturally disturbedforest that are maintained if 50% of the disturbed area is salvage logged. Source data are provided as a Source Data file.

Beta regression revealed that the proportion of a naturally disturbed area that needs to be left unlogged to maintain species richness did not change significantly with increasing time elapsed since disturbance (Fig. 5). This held true for both the retention area needed to maintain 90% of species unique to unlogged disturbed forest (estimated degrees of freedom of years since disturbance = 1.001, *p*-value = 0.11, adj. *r*² = 0.75), and the portion of species unique to unlogged naturally disturbed forest that remain after 50% of a given naturally disturbed area is salvage logged (estimated degrees of freedom of years since disturbance = 1.001, *p*-value = 0.13, adj. *r*² = 0.75).

## Discussion

Using a global multi-taxa dataset, we estimate retention benchmarks needed for biodiversity conservation in naturally disturbed forests. We find that, across all investigated disturbance types, an average of 75% of the disturbed area needs to be unlogged to maintain 90% of the disturbed forest’s unique species. The required areas to be left unlogged, however, varied considerably among taxonomic groups, with species groups associated with dead wood, such as saproxylic beetles and wood-inhabiting fungi, generally requiring larger portions (85%) than non-saproxylic taxa (72%). Our quantitative assessment has the additional advantage that it can be used to set any desired benchmark for biodiversity conservation. Our results also depict a relatively steep increase in species richness at the low part of portions of retained naturally disturbed area (Fig. 3).

Although comparisons with existing studies conducted under conventional logging schemes (i.e., without natural disturbances) are difficult, our results appear broadly similar to findings from work done in boreal forests of Finland^31^. There, wood amounts of 10 m³ and 50 m³ per ha were retained during clear-cut harvesting of pine-dominated stands of around 290 m³ per ha, corresponding to 3 and 17% of the original wood volume, respectively. Even the 3% retention level harbored many rare and red-listed saproxylic beetles, highlighting the importance of retaining even small amounts of wood^31^. Our results for saproxylic species are consistent with the conceptual framework developed by Fedrowitz et al. ^32^, which predicted an increase in forest species and a decline in open-habitat species (Fig. 4). This finding is not surprising because many positive effects of natural disturbances on the richness of forest specialists are related to an increase in available deadwood resources^7^. By contrast, taxonomic groups that typically reach high species richness in open areas needed, on average, lower portions of unlogged, disturbed area to be retained (Fig. 4); thus, epigeic spiders and ground beetles still maintained high proportions of species unique to unlogged forests even if 50% of the area was logged (Fig. 4b). In our data, these groups had high numbers of species in unlogged disturbed forests (Fig. 4, right column). Hence, the retention of smaller proportions of naturally disturbed forest might be sufficient to maintain a shady and moist microclimate, which can promote species associated with unlogged naturally disturbed forests (e.g., epigeic spiders and ground-dwelling beetles^33^). Finally, preservation of all species unique to unlogged naturally disturbed areas requires the retention of on average 100% of the disturbed area, whereas in some cases 100% richness could be reached with less than 100% retention (see standard deviation in Fig. 3a). This is particularly relevant for protected areas, where biodiversity protection is a primary aim^4^.

Despite the small amount of data available from insect-affected forests, they appeared to need slightly higher amounts of retention area needed than forests subject to other kinds of natural disturbance to maintain the same amount of species richness (Fig. 3). This could arise from differences in the biological legacies left behind after different types of disturbance. In contrast to wildfires or windstorms, insect outbreaks typically leave behind intact ground, understory and midstory vegetation, as well as a longer-lasting vertical structure (i.e., insect-killed trees)^34^, and thus logging likely results in a greater degree of environmental impact.

Our values for retention benchmarks did not change over time (Fig. 5), indicating that the importance of retained areas did not decrease or increase over the course of succession within the first ca. 20 years after salvage logging, which was the period best covered by our data. This time span is shorter than cutting cycles in most of the investigated forest types, which range between 60 and 120 years, but covers the most significant changes in tree cover and deadwood amount during the first 100 years. Differences in taxonomic, functional and phylogenetic diversity of bird communities sampled in unlogged disturbed plots and salvage-logged plots can persist or even increase over 17 years following fire and wind disturbance^11^. Similarly, a multi-taxa approach, including vascular plants, bryophytes, lichens, wood-inhabiting fungi, saproxylic beetles, and birds, revealed a limited change in dissimilarities between unlogged disturbed plots and salvage-logged plots over the first seven years of succession^30^. In that study, the remaining dissimilarities in communities were caused primarily by the presence or absence of rare species^30^, quantified based on a similar statistical framework as in our study. Our results therefore imply that the positive effect of retention during salvage logging on biodiversity remains over the course of early succession. However, in some cases forests might need several centuries to regrow key structures—for instance, to recover the availability of tree hollows—so that the impacts of salvage logging on biodiversity can exceed 100 years^35^.

Our statistical approach can be applied to any combination of two types of land use or habitats to reveal benchmarks for optimal enhancing overall biodiversity. This approach considers beta diversity by addressing the species unique to unlogged areas while simultaneously accounting for species that occur in both logged and unlogged areas within a landscape (Fig. 1). The detection of shared species is important, since many altered habitats typically share a large portion of species with the original habitat. Hence, comparisons based on alpha diversity alone might lead to biased benchmarks, since the net change in species richness can be small while the turnover between communities can be large^29^. This becomes particularly relevant for species unique to early-successional stages of naturally disturbed forests, where salvage logging can lead to marked changes in communities despite limited changes in alpha diversity of some taxonomic groups^7,34^. Contrarily, changes in species richness might be large while the turnover between communities in different habitat types is relatively small, i.e., a high number of species is shared^36^. Shared species can include species that utilize both forest types, for instance by roosting or breeding in unlogged disturbed forest and foraging in both unlogged and salvage-logged forests^37,38^.

The benchmarks reported in our study are based on the number of species unique to unlogged, naturally disturbed forests. Hence, the overall increase or decrease in species richness with increasing extent of salvage logging depends both on the loss of species unique to unlogged naturally disturbed forests and on the simultaneous colonization of species typically found in salvage-logged forests^27^. Since shared species richness varies little across different proportional mixtures of two habitat types in statistical simulations^27^, maintaining a minimum number of unique species from one of the two habitat types, i.e., unlogged naturally disturbed forest in our case, is approximately equivalent to maintaining a specific level of overall species richness. This pattern underlines the generalizability of our results, providing evidence-based benchmarks to protect biodiversity in naturally disturbed forests.

## Methods

### Database

We compiled a global database of species abundances in salvage-logged and unlogged naturally disturbed plots by extending two recent reviews (Fig. 2)^6,7^. The data compilation followed a systematic review protocol to ensure high quality standards in data selection^39^. We retained only those datasets that were based on field surveys and excluded modeling studies. In addition to the use of raw data from published studies, we extended three of the studies^40–42^ by conducting additional surveys, adhering, in each case, to the original sampling design (Supplementary Table 1). All studies had to be conducted in forests where more than 75% of the trees had been affected by wildfires, insect outbreaks, or windstorms. Each study could provide multiple entries in our database given the number of investigated years and taxonomic groups. Study designs needed to provide comparisons between completely salvage-logged plots and completely unlogged, naturally disturbed reference plots, and both treatments had to be properly replicated^43^. The plots sampled in both treatments had to be located in the same forest affected by the same disturbance event, of similar size, and surveyed with the same sampling effort^7^. All study designs were checked for spatial autocorrelation between plots of the same treatment and excluded if necessary. Salvage logging had to have taken place in less than 36 months following the natural disturbance event.

The final database included full species-by-plot abundance matrices of bats^44^, birds^12,40–42,45–51^, ground beetles (Coleoptera, Carabidae)^52,53^, deadwood dependent (i.e., saproxylic) beetles^8,42^, non-saproxylic beetles^42^, Hymenoptera^42^, epigeic spiders^53^, epigeic and epixylic bryophytes^42,54^, epigeic and epixylic lichens^42^, hoverflies^53^, nocturnal moths^55^, scuttle flies^56^, true bugs (Heteroptera)^53^, wood-inhabiting fungi^42^, and vascular plants^13,42,55,57–62^. We defined deadwood-dependent beetles, epixylic lichens, epixylic bryophytes, and wood-inhabiting fungi as saproxylic species groups. The database included the variables disturbance type, time elapsed since disturbance, and taxonomic group, which we used as covariates in our analysis. Our database consisted of 201 individual species matrices distributed across 17 taxonomic groups from studies conducted predominantly in temperate and boreal forests for up to 34 years following natural disturbance events (Fig. 2).

### Data analysis

We used a statistical approach that extends classical rarefaction and extrapolation^63^ towards a proportional mixture of two rarefaction/extrapolation curves derived from two distinct assemblages^27^. The analyses were conducted following the R code miNEXT (mixed iNterpolation/EXTrapolation, available at https://github.com/AnneChao).

The conventional species-area relationship describes the relationship between species richness and the sampling area using a parametric function (such as the Arrhenius model or Gleason model). However, a specified parametric function cannot be applied to all types of data. In our case, a within-habitat rarefaction/extrapolation curve represents a non-parametric species-area relationship estimated from the data themselves (Fig. 1, dashed curves). Estimated non-parametric species-area relationships can be applied to all types of data and compared across studies. Our proportional mixture enables the quantification of the between-habitat species compositional difference (i.e., beta diversity), which can be incorporated in the analysis to predict the resulting diversity change due to salvage logging^27^.

Mixed rarefaction/extrapolation can either be applied to species abundances or species incidence/occurrence frequencies among plots. Furthermore, it can be applied even to unbalanced study designs, i.e., when the number of salvage-logged plots and disturbed unlogged plots differ. Our mixed rarefaction/extrapolation was based on *T*_1_ plots surveyed in unlogged, disturbed forest and *T*_2_ plots surveyed in corresponding salvaged logged forests. For all data, we treated the number of occurrences of each species among multiple plots as a proxy for the abundance of that species, as multiple incidence data are less sensitive than abundance data to possible clustering or aggregation of individuals^64^. When a proportion of unlogged disturbed plots (e.g., *t* plots) are salvage logged, it is equivalent to replacing these *t* plots by the same number of plots randomly selected from salvaged logged forests. Using a mixture of rarefaction and extrapolation, we can analytically retrieve the species richness of the mixed assemblage (Fig. 1).

Mixed rarefaction/extrapolation is independent of the underlying spatial arrangement of the study plots, i.e., it is based on comparisons of plots randomly selected from any location of a study design and is independent of plot size and the number of plots within a respective study, as long as all plots within a study are of similar sizes. Our benchmarks are hence independent of the spatial arrangement of the underlying study plots^27^. This is particularly important as detailed information about the size of a disturbed area for each study year was not available. Mixed rarefaction/extrapolation also provides species composition information, i.e., shared species richness and the richness of species that are unique to either unlogged, disturbed or salvage-logged plots under any proportion of the mixture^27^. In our synthesis, we focused on the richness of species unique to unlogged, disturbed forests (Fig. 1). Mixed rarefaction/extrapolation allows for the estimation of the number of plots associated with a specific level of species richness that is unique to unlogged, disturbed plots. The proportion of these plots can subsequently be used as a proxy for the proportion of area that needs to be excluded from salvage logging^27^. Using mixed rarefaction/extrapolation, we estimated retention areas for different taxonomic groups and disturbance types to identify benchmarks of group-specific salvage-logging retention.

Finally, we fitted beta regressions by means of the function *gam* with family *betar* in the R-package *mgcv*^65^ to test the effect of time since disturbance on retention benchmarks. For this purpose, we selected the year since disturbance as smooth term to account for possible non-linear relationships^11^. Furthermore, we controlled for study identity, taxonomic group, and disturbance type via additional predictors.

### Reporting summary

Further information on research design is available in the Nature Research Reporting Summary linked to this article.

## Supplementary information

Supplementary Information

Reporting Summary

## Data Availability

The data collected in the Bavarian Forest National Park may be found in the BIOtime (http://biotime.st-andrews.ac.uk/downloadArea.php) database^66^. All other original data underlying our analyses can be made available by the respective co-authors upon reasonable request. Source data are provided with this paper.

## Source data

Source Data
