# Supplementary material for: Estimating retention benchmarks for salvage logging to protect biodiversity
Source: Nat Commun. 2020 Sep 21;11:4762. doi: 10.1038/s41467-020-18612-4 (PMC7505835; doi:10.1038/s41467-020-18612-4)
Supplement: Supplementary file 1 — Supplementary Information [file 41467_2020_18612_MOESM1_ESM.docx]

Supplementary Information File

**Estimating retention benchmarks for salvage logging to protect biodiversity**

Thorn et al.

Supplementary Table 1. Summary of the data included in the study. Note that plot sizes refer to the size of plots given the respective references. Note that ‘not applicable’ (n.a.) is given, if the respective surveys were conducted on a standardized number of deadwood objects (e.g. wood-inhabiting fungi) or used insect traps, where the range cannot be standardized (e.g. flight-interception traps, pitfall-traps).

| **Taxonomic group** | **Location** | **Disturbance** | **Number of studied years** | **First studied year after disturbance** | **Last studied year after disturbance** | **Number of disturbed unlogged plots** | **Number of salvage- logged plots** | **Single plot size [ha]** | **Ref.** |
| --- | --- | --- | --- | --- | --- | --- | --- | --- | --- |
| Vascular plants | Oregon, USA | Fire | 1 | 4 | 4 | 80 | 85 | 0.0013-0.09 | ^1^ |
| Birds | Oregon, USA | Fire | 1 | 4 | 4 | 64 | 58 | 1 | ^2^ |
| Birds | Oregon, USA | Fire | 2 | 1 | 2 | 5 | 5 | 2.01 | ^3^ |
| ^S^Saproxylic beetles | Alberta, Canada | Fire | 2 | 1 | 2 | 6 | 6 | n.a. | ^4^ |
| Birds | Montana, USA | Fire | 18 | 1 | 27 | 6–2410 | 8–651 | 3.14 | ^5^* |
| Ground beetles | Alberta, Canada | Fire | 1 | 2 | 2 | 48 | 48 | n.a. | ^6^ |
| Vascular plants | Alberta, Canada | Fire | 2 | 2 | 34 | 38–40 | 40–45 | 0.0004 | ^7^ |
| Vascular plants | Alberta, Canada | Fire | 1 | 2 | 2 | 13 | 14 | 0.0004 | ^8^ |
| Vascular plants | Colorado, USA | Insect outbreak | 1 | 2 | 2 | 5 | 5 | 0.005 | ^9^ |
| Vascular plants | Quebec, Canada | Wind | 1 | 4 | 4 | 12 | 24 | 0.0004 | ^10^ |
| Epigeic bryophytes | Canary Islands, Spain | Fire | 1 | 3 | 3 | 86^m^ | 73^m^ | 0.01 | ^11^ |
| Birds | Sierra Nevada, Spain | Fire | 2 | 2 | 3 | 9 | 18 | 2.7 | ^12^ |
| Vascular plants | Sierra Nevada, Spain | Fire | 1 | 2 | 2 | 9 | 9 | 2.7 | ^13^ |
| Birds | Catalonia, Spain | Fire | 2 | 2 | 3 | 15–42 | 16–43 | 3.7 | ^14^ |
| Epigeic spiders | Switzerland | Wind | 7 | 1 | 19 | 8–13 | 8–10 | n.a. | ^15^ |
| Ground beetles | Switzerland | Wind | 7 | 1 | 19 | 8–13 | 8–10 | n.a. | ^15^ |
| True bugs | Switzerland | Wind | 7 | 1 | 19 | 6–8 | 6 | n.a. | ^15^ |
| Hoverflies | Switzerland | Wind | 7 | 1 | 19 | 6–8 | 6 | n.a. | ^15^ |
| Bats | Bavarian Forest National Park, Germany | Insect outbreak | 1 | 5 | 5 | 8 | 8 | n.a. | ^16^ |
| Vascular plants | Bavarian Forest National Park, Germany | Insect outbreak | 1 | 5 | 5 | 8 | 8 | 0.785 | ^17^ |
| Nocturnal moths | Bavarian Forest National Park, Germany | Insect outbreak | 1 | 5 | 5 | 8 | 8 | n.a. | ^17^ |
| Vascular plants | Bavarian Forest National Park, Germany | Wind | 7 | 1 | 11 | 23–24 | 19–20 | 0.02 | ^18^* |
| Epigeic bryophytes | Bavarian Forest National Park, Germany | Wind | 6 | 1 | 11 | 24–25 | 24 | 0.02 | ^18^* |
| ^S^Epixylic bryophytes | Bavarian Forest National Park, Germany | Wind | 6 | 1 | 11 | 19 | 18–19 | n.a. | ^18^* |
| ^S^Epigeic lichens | Bavarian Forest National Park, Germany | Wind | 6 | 1 | 11 | 3–16 | 2–23 | 0.02 | ^18^* |
| ^S^Epixylic lichens | Bavarian Forest National Park, Germany | Wind | 6 | 1 | 11 | 19 | 18–19 | n.a. | ^18^* |
| ^S^Wood-inhabiting fungi | Bavarian Forest National Park, Germany | Wind | 6 | 1 | 11 | 19 | 11–19 | n.a. | ^18^* |
| ^S^Saproxylic beetles | Bavarian Forest National Park, Germany | Wind | 5 | 1 | 11 | 22 | 22 | n.a. | ^18^* |
| Birds | Bavarian Forest National Park, Germany | Wind | 4 | 1 | 11 | 20–21 | 20–32 | 0.785 | ^18^* |
| Hymenoptera | Bavarian Forest National Park, Germany | Wind | 1 | 11 | 11 | 22 | 22 | n.a. | ^18^* |
| Non-saproxylic beetles | Bavarian Forest National Park, Germany | Wind | 1 | 11 | 11 | 22 | 22 | n.a. | ^18^* |
| Birds | Sweden | Fire | 4 | 1 | 4 | 69 | 40 | 0.785 | ^19^* |
| Scuttle flies | Poland | Wind | 1 | 3 | 3 | 6 | 5 | n.a. | ^20^ |
| Birds | Poland | Wind | 3 | 5 | 11 | 38–49 | 54–57 | 3.14 | ^21^* |
| Birds | South Korea | Fire | 1 | 5 | 5 | 19 | 19 | 1 | ^22^ |
| Birds | South Korea | Fire | 2 | 7 | 8 | 10–14 | 10–14 | 10 | ^23^ |
| Birds | Victoria, Australia | Fire | 6 | 1 | 7 | 18-42 | 24-42 | 0.785 | ^24^ |
| Vascular plants | Victoria, Australia | Fire | 1 | 2 | 2 | 28 | 28 | 0.03 | ^25^ |

^S^ indicates saproxylic species groups

^m^ indicates that microplots where used for the analyses

* The study dataset was expanded by adding unpublished data

**References**

1. Donato, D. C., Fontaine, J. B., Kauffman, J. B., Robinson, D. & Law, B. E. Fuel mass and forest structure following stand-replacement fire and post-fire logging in a mixed-evergreen forest. *Int. J. Wildl. Fire* **22**, 652–666 (2013).

2. Fontaine, J. B., Donato, D. C., Robinson, W. D., Law, B. E. & Kauffman, J. B. Bird communities following high-severity fire: Response to single and repeat fires in a mixed-evergreen forest, Oregon, USA. *For. Ecol. Manage.* **257**, 1496–1504 (2009).

3. Cahall, R. E. & Hayes, J. P. Influences of postfire salvage logging on forest birds in the Eastern Cascades, Oregon, USA. *For. Ecol. Manage.* **257**, 1119–1128 (2009).

4. Cobb, T. P. *et al.* Effects of postfire salvage logging on deadwood-associated beetles. *Conserv. Biol.* **25**, 94–104 (2011).

5. Hutto, R. L. & Young, J. Regional landbird monitoring: perspectives from the Northern Rocky Mountains. *Wildl. Soc. Bull.* **30**, 738–750 (2002).

6. Koivula, M. & Spence, J. R. Effects of post-fire salvage logging on boreal mixed-wood ground beetle assemblages (Coleoptera, Carabidae). *For. Ecol. Manage.* **236**, 102–112 (2006).

7. Kurulok, S. E. & Macdonald, S. E. Impacts of postfire salvage logging on understory plant communities of the boreal mixedwood forest 2 and 34 years after disturbance. *Can. J. For. Res.* **37**, 2637–2651 (2007).

8. Macdonald, S. E. Effects of partial post-fire salvage harvesting on vegetation communities in the boreal mixedwood forest region of northeastern Alberta, Canada. *For. Ecol. Manage.* **239**, 21–31 (2007).

9. Fornwalt, P. J. *et al.* Short-term understory plant community responses to salvage logging in beetle-affected lodgepole pine forests. *For. Ecol. Manage.* **409**, 84–93 (2018).

10. Waldron, K., Ruel, J.-C., Gauthier, S., De Grandpré, L. & Peterson, C. J. Effects of post-windthrow salvage logging on microsites, plant composition and regeneration. *Appl. Veg. Sci.* **17**, 323–337 (2014).

11. Hernández-Hernández, R., Castro, J., Del Arco Aguilar, M., Fernández-López, Á. B. & González-Mancebo, J. M. Post-fire salvage logging imposes a new disturbance that retards succession: The case of bryophyte communities in a Macaronesian laurel forest. *Forests* **8**, 1–16 (2017).

12. Castro, J., Moreno-Rueda, G. & Hódar, J. Experimental test of postfire management in pine forests: impact of salvage logging versus partial cutting and nonintervention on bird-species assemblages. *Conserv. Biol.* **24**, 810–819 (2010).

13. Leverkus, A. B., Lorite, J., Navarro, F. B., Sánchez-Cañete, E. P. & Castro, J. Post-fire salvage logging alters species composition and reduces cover, richness, and diversity in Mediterranean plant communities. *J. Environ. Manage.* **133**, 323–31 (2014).

14. Rost, J., Clavero, M., Brotons, L. & Pons, P. The effect of postfire salvage logging on bird communities in Mediterranean pine forests: the benefits for declining species. *J. Appl. Ecol.* **49**, 644–651 (2012).

15. Wermelinger, B. *et al.* Impact of windthrow and salvage-logging on taxonomic and functional diversity of forest arthropods. *For. Ecol. Manage.* **391**, 9–18 (2017).

16. Mehr, M., Brandl, R., Kneib, T. & Müller, J. The effect of bark beetle infestation and salvage logging on bat activity in a national park. *Biodivers. Conserv.* **21**, 2775–2786 (2012).

17. Thorn, S. *et al.* Guild-specific responses of forest Lepidoptera highlight conservation-oriented forest management – implications from conifer-dominated forests. *For. Ecol. Manage.* **337**, 41–47 (2015).

18. Thorn, S. *et al.* Changes in the dominant assembly mechanism drive species loss caused by declining resources. *Ecol. Lett.* **19**, 163–170 (2016).

19. Zmihorski, M. *et al.* Early post-fire bird community in European boreal forest: Comparing salvage-logged with non-intervention areas. *Glob. Ecol. Conserv.* **18**, e00636 (2019).

20. Durska, E. Effects of disturbances on scuttle flies (Diptera: Phoridae) in Pine Forests. *Biodivers. Conserv.* **22**, 1991–2021 (2013).

21. Zmihorski, M. The effect of windthrow and its management on breeding bird communities in a managed forest. *Biodivers. Conserv.* **19**, 1871–1882 (2010).

22. Choi, C. Y., Lee, E. J., Nam, H. Y. & Lee, W. S. Effects of postfire logging on bird populations and communities in burned forests. *Jorunal Korean For. Soc.* **96**, 115–123 (2007).

23. Lee, E.-J., Lee, W.-S., Son, S. H. & Rhim, S.-J. Differences in bird communities in postfire silvicultural practices stands within pine forest of South Korea. *Landsc. Ecol. Eng.* **7**, 137–143 (2011).

24. Lindenmayer, D. B., Mcburney, L., Blair, D., Wood, J. & Banks, S. C. From unburnt to salvage logged: Quantifying bird responses to different levels of disturbance severity. *J. Appl. Ecol.* 1626–1636 (2018). doi:10.1111/1365-2664.13137

25. Blair, D. P., McBurney, L. M., Blanchard, W., Banks, S. C. & Lindenmayer, D. B. Disturbance gradient shows logging affects plant functional groups more than fire. *Ecol. Appl.* **26**, 2280–2301 (2016).
