# Supplementary material for: Estimating retention benchmarks for salvage logging to protect biodiversity
Source: Nat Commun. 2020 Sep 21;11:4762. doi: 10.1038/s41467-020-18612-4 (PMC7505835; doi:10.1038/s41467-020-18612-4)
Supplement: Supplementary file 2 — Reporting Summary [file 41467_2020_18612_MOESM2_ESM.pdf]

nature research

Corresponding author(s): Simon Thorn

Last updated by author(s): 26.07.2020

Reporting Summary

Nature Research wishes to improve the reproducibility of the work that we publish. This form provides structure for consistency and transparency in reporting. For further information on Nature Research policies, see Authors & Referees and the Editorial Policy Checklist .

Statistics

For all statistical analyses, confirm that the following items are present in the figure legend, table legend, main text, or Methods section.

n/a	Confirmed	
☐	☒	The exact sample size (*n*) for each experimental group/condition, given as a discrete number and unit of measurement
☐	☒	A statement on whether measurements were taken from distinct samples or whether the same sample was measured repeatedly
☐	☒	The statistical test(s) used AND whether they are one- or two-sided<br>*Only common tests should be described solely by name; describe more complex techniques in the Methods section.*
☐	☒	A description of all covariates tested
☐	☒	A description of any assumptions or corrections, such as tests of normality and adjustment for multiple comparisons
☐	☒	A full description of the statistical parameters including central tendency (e.g. means) or other basic estimates (e.g. regression coefficient) AND variation (e.g. standard deviation) or associated estimates of uncertainty (e.g. confidence intervals)
☐	☒	For null hypothesis testing, the test statistic (e.g. *F*, *t*, *r*) with confidence intervals, effect sizes, degrees of freedom and *P* value noted<br>*Give P values as exact values whenever suitable.*
☐	☒	For Bayesian analysis, information on the choice of priors and Markov chain Monte Carlo settings
☐	☒	For hierarchical and complex designs, identification of the appropriate level for tests and full reporting of outcomes
☐	☒	Estimates of effect sizes (e.g. Cohen's *d*, Pearson's *r*), indicating how they were calculated

Our web collection on statistics for biologists contains articles on many of the points above.

Software and code

Policy information about availability of computer code

Data collection	No software was used to collect the data.
Data analysis	All analyses were conducted with the free statistical software R. The codes are available online at https://github.com/AnneChao. We used the R package mgcv 1.8-31.

For manuscripts utilizing custom algorithms or software that are central to the research but not yet described in published literature, software must be made available to editors/reviewers. We strongly encourage code deposition in a community repository (e.g. GitHub). See the Nature Research guidelines for submitting code & software for further information.

Data

Policy information about availability of data

All manuscripts must include a data availability statement. This statement should provide the following information, where applicable:

- Accession codes, unique identifiers, or web links for publicly available datasets
- A list of figures that have associated raw data
- A description of any restrictions on data availability

Data are made available by original data owners upon reasonable request.

Field-specific reporting

Please select the one below that is the best fit for your research. If you are not sure, read the appropriate sections before making your selection.

☐ Life sciences ☐ Behavioural & social sciences ☒ Ecological, evolutionary & environmental sciences

Ecological, evolutionary & environmental sciences study design

All studies must disclose on these points even when the disclosure is negative.

Study description	We compiled a global database of species found in salvage logged and unlogged plots by extending two recent reviews. The data compilation followed a systematic review protocol to ensure high standards in data selection.
Research sample	Our database consisted of 201 individual species matrices distributed across 14 taxonomic groups from studies conducted across the Northern Hemisphere for up to 34 years following natural disturbances. We compiled a global database of species found in salvage logged and unlogged naturally disturbed plots by extending two recent reviews. The data compilation followed a systematic review protocol to ensure high quality standards in data selection. We retained only those datasets based on field surveys and excluded modelling studies. In addition to the use of the raw data from published studies, we extended three of the studies by conducting additional surveys, adhering, in each case, to the original sampling design
Sampling strategy	We collected all available data sources for our synthesis following a systematic review protocol and two recent reviews. The database sufficiently covers the current state of knowledge on the effects of salvage logging on biodiversity.
Data collection	All studies had to be conducted in forests where more than 75% of the trees had been affected by wildfires, insect outbreaks, or wind-storms. Each study can provide multiple entries in our database given the number of investigated years and taxonomic groups. Study designs needed to provide comparisons between completely salvage logged plots and completely unlogged control plots, and both treatments had to be properly replicated (for details see Leverkus et al., 2018b). The plots sampled in both treatments had to be located in the same forest affected by the same disturbance event, of similar size, and surveyed with the same sampling effort6. Salvage logging had to have taken place less than 36 months following the natural disturbance. The final database includes full species-by-plot abundance matrices of bats, birds, ground beetles (Coleoptera, Carabidae), dead-wood dependent (i.e. saproxylic) beetles, non-saproxylic beetles, Hymenoptera, epigeic spiders, epigeic and epixylic bryophytes, epigeic and epixylic lichens, hoverflies, nocturnal moths, scuttle flies, true bugs (Heteroptera), wood-inhabiting fungi, and vascular plants. We defined dead-wood dependent beetles, epixylic lichens, epixylic bryophytes, and wood-inhabiting fungi as saproxylic species. The database included the variables disturbance type and taxonomic group, which we used as covariates in our analysis.
Timing and spatial scale	Data were taken worldwide within the last 30 years.
Data exclusions	Study designs needed to provide comparisons between completely salvage logged plots and completely unlogged control plots, and both treatments had to be properly replicated (for details see Leverkus et al., 2018b). The plots sampled in both treatments had to be located in the same forest affected by the same disturbance event, of similar size, and surveyed with the same sampling effort6. Salvage logging had to have taken place less than 36 months following the natural disturbance. All other studies were excluded.
Reproducibility	Our synthesis is based on published studies. We provide 95% confidence bands obtained by 200 randomizations each along with our results.
Randomization	The final database includes full species-by-plot abundance matrices of bats, birds, ground beetles (Coleoptera, Carabidae), dead-wood dependent (i.e. saproxylic) beetles, non-saproxylic beetles, Hymenoptera, epigeic spiders, epigeic and epixylic bryophytes, epigeic and epixylic lichens, hoverflies, nocturnal moths, scuttle flies, true bugs (Heteroptera), wood-inhabiting fungi, and vascular plants. We defined dead-wood dependent beetles, epixylic lichens, epixylic bryophytes, and wood-inhabiting fungi as saproxylic species. The database included the variables disturbance type and taxonomic group, which we used as covariates in our analysis.
Blinding	Blinding is not possible in setting up insect traps, doing bird monitoring, and vegetation surveys.

Did the study involve field work? ☐ Yes ☒ No

Reporting for specific materials, systems and methods

We require information from authors about some types of materials, experimental systems and methods used in many studies. Here, indicate whether each material, system or method listed is relevant to your study. If you are not sure if a list item applies to your research, read the appropriate section before selecting a response.

Materials & experimental systems

n/a	Involved in the study	
☒	☐	Antibodies
☒	☐	Eukaryotic cell lines
☒	☐	Palaeontology
☒	☐	Animals and other organisms
☒	☐	Human research participants
☒	☐	Clinical data

Methods

n/a	Involved in the study	
☒	☐	ChIP-seq
☒	☐	Flow cytometry
☒	☐	MRI-based neuroimaging
